# The Hispanic Paradox and Older Adults’ Disabilities: Is There a Healthy Migrant Effect? 

**DOI:** 10.3390/ijerph10051786

**Published:** 2013-05-03

**Authors:** Esme Fuller Thomson, Amani Nuru-Jeter, Dawn Richardson, Ferrah Raza, Meredith Minkler

**Affiliations:** 1 Factor-Inwentash Faculty of Social Work, University of Toronto 246 Bloor Street West, Toronto, ON M5S 1A1, Canada; E-Mail: ferrah.raza@spi.ox.ac.uk; 2 School of Public Health, University of California Berkeley, CA 94720, USA; E-Mails: anjeter@berkeley.edu (A.N.-J.); mink@berkeley.edu (M.M.); 3 College of Urban and Public Affairs, Portland State University, Portland, OR 97201, USA; E-Mail: drichar2@pdx.edu

**Keywords:** Hispanic paradox, healthy migrant effect, salmon effect, reverse migration, functional limitations, disability, immigration, Mexican Americans

## Abstract

The “Hispanic Paradox” suggests that despite rates of poverty similar to African Americans, Hispanics have far better health and mortality outcomes, more comparable to non-Hispanic White Americans. Three prominent possible explanations for the Hispanic Paradox have emerged. The “Healthy Migrant Effect” suggests a health selection effect due to the demands of migration. The Hispanic lifestyle hypothesis focuses on Hispanics’ strong social ties and better health behaviors. The reverse migration argument suggests that the morbidity profile in the USA is affected when many Hispanic immigrants return to their native countries after developing a serious illness. We analyzed data from respondents aged 55 and over from the nationally representative 2006 American Community Survey including Mexican Americans (13,167 U.S. born; 11,378 immigrants), Cuban Americans (314 U.S. born; 3,730 immigrants), and non-Hispanic White Americans (629,341 U.S. born; 31,164 immigrants). The healthy migrant effect was supported with SES-adjusted disability comparable between Mexican, Cuban and non-Hispanic Whites born in the USA and all immigrants having lower adjusted odds of functional limitations than U.S. born non-Hispanic Whites. The reverse migration hypothesis was partially supported, with citizenship and longer duration in the USA associated with higher rates of SES-adjusted disability for Mexican Americans. The Hispanic healthy life-style explanation had little support in this study. Our findings underline the importance of considering nativity when planning for health interventions to address the needs of the growing Hispanic American older adult population.

## 1. Introduction

Over the past two decades a growing body of research has suggested that there is an “Hispanic Paradox” in the United States. Although racial/ethnic disparities in health have been well documented, with racial minority Americans disproportionately burdened by poor health outcomes [1,2], certain patterns of health outcomes among the Hispanic population challenge our understanding of the relationship between the social gradient and health. Despite disadvantaged social position (including education and income), Hispanics have better than expected health outcomes in such key areas as life expectancy and infant mortality [2] although the Hispanic paradox has not been shown with all diseases [3] or ages [4,5]. The life expectancy data are particularly supportive of the Hispanic Paradox. In a review of the literature, Franzini and colleagues found evidence for the Hispanic mortality paradox [6] demonstrating that among Hispanics, mortality rates are lower than among non-Hispanic Whites in general, despite lower than average income and educational levels [7]. Although some of this likely reflected data discrepancies and the fact that the term Hispanic was not always clearly defined [6,8], the findings overall were suggestive of the paradox effect. In the United States, Hispanics’ life expectancy at birth is 2.5 years longer than that of non-Hispanic Whites and 7.7 years longer than Blacks (80.6 years, 78.1 years and 72.9 years, respectively) [9]. Additional studies have found that among adults ages 45–64 and 65-plus, foreign-born Mexican Americans have lower mortality rates than native-born non-Hispanic Whites [7,9,10,11,12]. 

Three salient explanations of the Hispanic Paradox have emerged from the literature, which will be discussed later. With a rapidly aging population, and what Hayes-Bautista [13] has referred to as “the browning of the graying” of the United States, there is a need to expand the foci of research on the Hispanic Paradox beyond discrete disease outcomes or all cause mortality to investigate disability as a measure of quality of life indicative of overall well-being among older adults. This is especially salient given the aging of the U.S. population. The present study used data from a 2006 nationally representative survey of community dwelling older adults, comparing functional limitation rates among six groups (U.S. born non-Hispanic Whites, U.S. born Mexican Americans, and U.S. born Cuban Americans, non-Hispanic White immigrants, Mexican American immigrants, Cuban immigrants). Following a brief overview of the demographics of the American Hispanic population, we briefly summarize the leading hypotheses used to explain the Hispanic paradox. Next, we describe the objectives and methods of the current study, which aims to examine the “healthy migrant effect” hypothesis with respect to rates of functional limitations in older Hispanic and non-Hispanic Whites. We further identify factors associated with resiliency among Hispanic Americans. 

### 1.1. Hispanics in the United States

In 2010 there were 50.5 million people of Hispanic or Latino origin in the U.S., accounting for 16% of the population, [14] and making them second only to Caucasians in number [15]. Demographic projections show that the Hispanic population will triple in size by 2050, resulting in Hispanics comprising almost one-third of the entire U.S. population [16]. From 2000 to 2006, the Hispanic growth rate was more than three times the growth rate of the overall U.S. population (24.3% compared to 6.1%) [17] and accounted for over half of the population growth in the U.S. since 2000. Although high birth rates and low death rates among this group accounted for most of the increased Hispanic growth rate [15], immigration also played a substantial role, with just under 40% of all Hispanics in the U.S. being foreign-born [18]. Older individuals form a far smaller proportion of the Hispanic population. Just 6.2% of Hispanics in the U.S. in 2009 were aged 55–64, dropping to just 2.4% of those aged 75+ [19]. 

Despite these current low figures, the population of older Hispanic Americans is expected to more than double by 2050 [16]. The projected aging of the Hispanic population underscores the need for further research on the relevance of hypotheses such as the “healthy migrant effect” on current and future generations, particularly with respect to such outcomes as functional limitations, a robust and qualitative indicator of quality of life that goes beyond case definitions of disease entities to reflect the effect of health conditions or disease states on overall functioning. The disablement process suggests progression from pathology, impairment, functional limitations, and disability. We intentionally use “functional limitations” as it is a key component of the disablement process [20]. Although functional limitations represent significant impairment, it is less severe and affects a larger proportion of the older adult population relative to actual disability (e.g., limitations in Activities of Daily Living, such as eating or bathing).

### 1.2. Three Possible Explanations for the Hispanic Paradox

Three prominent possible explanations of the Hispanic paradox have emerged: the healthy migrant effect; Hispanic lifestyle and health behaviors; and reverse migration. 

#### 1.2.1. Healthy Migrant Effect

The “Healthy Migrant Effect” suggests that there is a healthy selection effect due to the substantial physical and mental demands of migration. Migrants are thought to be healthier than those who do not migrate from their country of origin [10,12,15]. Evidence in support of this explanation includes data demonstrating that immigrants have lower mortality rates in their host countries than the overall mortality rates of their countries of origin [10]. Crimmins and her colleagues further found that Mexican immigrants who remain in the U.S. are taller that non-migrants in Mexico and return migrants of the same age, suggesting that successful migrants had better childhood nutrition and overall health [21]. Crimmins *et al.* [22] subsequently reported support for migrant health selectivity using biological risk profiles such as blood pressure, blood glucose, and cholesterol showing that after adjustments for income and education, U.S. born Hispanics had higher risk but Hispanics immigrants were comparable to non-Hispanic White Americans. Yet even these findings are not definitive. 

Of particular salience to the present study is the suggestion that there is something about the *type of individuals* who choose to migrate and/or the *experience of migration itself* that is associated with hardiness in immigrants, which may in turn lead them to have better health outcomes than individuals born in the U.S. with comparable socioeconomic position (SEP) [10]. However, the particularly robust nature of new immigrants’ health due to the protective effect of self-selection and required health screening is posited to decrease with time after immigration due to acculturation stress, as well as adoption of unhealthy behaviors [23]. Although, to our knowledge, this has not yet been examined, we hypothesize that if self-selection is important, those who come in their childhood, probably under the volition of their parents, may be less healthy than adults who choose to immigrate.

#### 1.2.2. Hispanic Lifestyle and Health Behaviors

The Hispanic lifestyle explanation rests on the argument that social and cultural factors provide a protective effect for Hispanics with regard to mortality [7,10,12]. According to this hypothesis, the Hispanic mortality advantage is a result of strong social ties, more extensive social networks and healthier behaviors related to diet, smoking, alcohol consumption, and other health behaviors [10]. The evidence for this hypothesis is not conclusive: The diet of Hispanic American immigrants is healthier, on average, than that of Anglo-Americans [24]. However, the mortality advantage declines when barriers to accessing health services are considered, including financial constraints, and language and/or cultural differences. For instance, Hispanic women are less likely to use preventive health screening including breast examinations, mammograms, or Pap tests for a number of reasons, including both cultural norms and access factors [12,25,26]. As noted earlier, however, the mortality advantage does not apply to all diseases. For example, compared to non-Hispanic Whites, Hispanics have higher rates of mortality from diabetes [3,17].

Other research has shown that as Hispanic immigrants become acculturated to more Western lifestyle (*i.e.*, adopting the English language and American cultural values and practices), their protective health behaviors can in some cases, decline [27,28]. Lara *et al.* [27] note, however, that some of the changes that accompany more assimilation to mainstream American culture may be positive, (e.g., improved health care access) while others (e.g., exercise) remain inconclusive and still others are negative (e.g., diet [24]). Finally, Blue and Fenelon’s study of smoking in relation to the Hispanic paradox [29] suggests that, smoking accounts for over 75% of the difference in life expectancy at age 50 between U.S. Hispanics and non-Hispanic white men, with a rate discrepancy almost as marked among women. 

Although the smoking data are powerful, some cautions have been raised against employing explanations that solely or primarily rely on health behaviors and social networks. Viruell-Fuentes [30] argues that acculturation explanations focus our attention on the micro at the expense of the macro and in so doing limit our understanding of how individuals experience the contexts into which they immigrate. Such an orientation may lead to assumptions that an entire pan-ethnic group (Latinos) shares a culture that directly translates into behaviors, values, and beliefs that impact members’ health in similar ways, across all subgroups, time, and place. For example, smoking rates are significantly higher among Cuban Americans than among Mexican Americans [31], highlighting the importance of disaggregating the “Hispanic American” category into more meaningful sub-groups.

Issues of limited conceptualization and measurement of acculturation also have been raised, as has the fact that the impact of acculturation on “Latino” immigrant health has been found to differ significantly between younger and older cohorts [4,5].

#### 1.2.3. Reverse Migration or the Salmon Effect

Reverse migration or the “Salmon Effect” hypothesizes that Hispanic immigrants return to their native countries after growing older, retiring from work, or developing a serious illness [12,32]. As a result of these immigrants returning to their home countries, those deaths are not included in U.S. mortality rates, thereby lowering the prevalence of chronic illness, disability and mortality observed among Hispanics in U.S. data records. There is some evidence for the Salmon Effect. One study found that return migration explained a small but significant proportion of the Hispanic paradox in self-reported health outcomes [33]. Another study found strong support for the reverse migration effect, but only among foreign-born Mexicans, not among other foreign-born Hispanics [10]. Turra and Elo [34] found some evidence for the Salmon Effect using Social Security data, albeit not enough to explain the mortality advantage. Finally, additional studies examining this hypothesis did not find evidence to support it and concluded that the Salmon Effect could not explain the observed mortality advantage [12]. We intentionally chose to focus on Mexican Americans and Cuban Americans to investigate the salmon effect because, throughout the past half century, the option of reverse migration was available to Mexican Americans but much less likely for Cuban Americans. For Cuban immigrants, reverse migration is an unlikely explanation of the Hispanic Paradox in mortality [10]. For political reasons since the revolution in 1959, few individuals return to Cuba. Therefore, any advantage in disability status that Cuban immigrants have in comparison to non-Hispanic Whites, after controlling for income and other factors, cannot be attributed to reverse migration. Furthermore, with the exclusion of Puerto Ricans, who are not immigrants and therefore cannot shed light on the healthy migrant effect, Mexican Americans and Cuban Americans are the two largest Hispanic groups in the United States. A number or regions in both Central America and South America have had less stability during this time period, with reverse migration at some points being more constrained by political situations (e.g., Chile under Pinochet, Nicaragua during the civil war) than at other times. With such variability over time, immigrants from Central or South America are not the ideal groups to study in order to investigate the reverse migration hypotheses.

### 1.3. Logic of the Study

The present study focused primarily on the Healthy Migrant effect with respect to functional limitations in older Hispanic Americans for several reasons. We wanted an outcome that did not rely on medical diagnosis to address the potential variation in health care utilization between ethnic groups. Disparities between Hispanic and non-Hispanic White respondents in access to and utilization of health care services may result in under-reporting of physician-diagnosed conditions. Therefore, we used self-reported functional limitations. Although self-reported data has limitations including recall and reporting bias, this may more accurately reflect true functional limitation status than physician diagnosis. Additionally, functional limitations are relatively common among the older working population [35] and therefore provide a useful outcome variable. 

Our use of the extremely large 2006 American Community Survey (ACS) allowed us to delineate between two Hispanic groups: Mexican Americans, who are typically able to return to their country of origin and Cuban Americans who were extremely unlikely to during the time of this study. The large data set also allowed us to explore differences between immigrants and non-immigrants, as well as time since immigration within each ethnic group. All of these data are essential elements for untangling the role of acculturation and Hispanic lifestyle.

In this study, we investigate an overarching hypothesis regarding the existence of the Hispanic Paradox with respect to functional limitations, followed by three more nuanced hypotheses related to the three major theoretical explanations for the Paradox, as these apply to functional limitations. 

### 1.4. Hypotheses

(1) An Hispanic Paradox Exists For Functional Limitations. Hispanic Americans should have lower socio-economic position (SEP)-adjusted odds of functional limitations compared to non-Hispanic Whites.

(2) Hispanic Lifestyle Explanation: Hispanic Americans, whether immigrants or U.S. born, should have lower SEP-adjusted odds of functional limitations than non-Hispanic Whites and this association will be moderated by level of acculturation. 

(3) Healthy Migrant Explanation: Immigrants (Hispanic and White) should have lower SEP-adjusted disability rates than non-Hispanic Whites born in the U.S. Among immigrants, the protective effect of self-selection and health screening of new immigrants should decrease over time as immigrants acquire diseases in the post-immigration period. The positive effect of immigration should not be as evident for those who migrated as children, where the process of migration can be assumed to be instigated by the parents or guardians rather than the respondent. If the healthy migrant hypothesis is the main driver of the Hispanic paradox, then all citizens born in the U.S., regardless of ethnicity, should have comparable rates of functional limitations. 

(4) Reverse Migration: The health advantage of Hispanics is due to ill Hispanic immigrants returning home. Among Hispanic immigrants, Cubans, who at this writing generally cannot return to Cuba, will experience more disability than Mexican Americans. Mexican American immigrants who are citizens and those who have been in the U.S. longer will be less likely to return to Mexico and therefore will have higher rates of disability than non-citizens and those who immigrated more recently.

## 2. Methods

### 2.1. Data Source

We analyzed a subsample of the 2006 American Community Survey (ACS), a nationally representative survey of community-dwelling Americans with a 97.5% response rate [36,37] gathered by the U.S. Census Bureau. Our subsample consisted of respondents aged 55 and over and included 13,167 Mexican Americans born in the U.S. and 11,378 Mexican American immigrants; 314 Cuban Americans born in the U.S. and 3,730 Cuban immigrants; and 629,341 non-Hispanic White Americans born in the U.S. and 31,164 non-Hispanic White immigrants. Individuals of other ethnicities were excluded from this study. Among sampled households, one household respondent age 15 or above was selected at random to provide information on all members of the selected housing unit. 

### 2.2. Measures

#### 2.2.1. Outcome Variable

*Functional limitation status.* Respondents were asked if they had a long-lasting condition that “substantially limits one or more basic physical activities such as walking, climbing stairs, reaching, lifting, or carrying”. Responses are dichotomous indicating “yes” or “no”.

#### 2.2.2. Independent Variables

*Ethnicity*. We categorized ethnicity in two distinct ways: Ethnicity 1 disregarded nativity: Non-Hispanic White, Mexican Americans, and Cuban Americans. Ethnicity 2 included information on ethnicity and nativity resulting in 6 categories: Non-Hispanic White Americans born in U.S., Non-Hispanic White immigrants, Mexican Americans born in the U.S., Mexican Americans immigrants, Cuban Americans born in the U.S., Cuban American immigrants. The U.S. born category included those born abroad to U.S. citizen parent or parents.

*Decade of immigration* measured as before 1950, 1950–1959; 1960–1969; 1970–1979; 1980–1989; 1990–1999; and 2000 or later.

*Age at immigration* categorized as under 16, 16–19, 20 and over, 

Whether *English was spoken at home* was characterized as yes, no. 

#### 2.2.3. Control Variables:

Control variables included *age* (55–64, 65–74, 75–84, 85 or older), *gender, highest level of education completed* (no education or only some primary school, some high school, high school graduation, bachelors degree, graduate degree). *Household* *income* was grouped into 6 categories based on percent of the federal poverty line taking household size and composition into account: below poverty line, 100–199%, 200–299%, 300–399%, 400–499%, 500% or more. *Immigration status* (yes, no) and decade of immigration were combined into one variable—*Immigration history*—with 8 categories: born in the U.S., immigrated prior to 1950, immigrated 1950–1959; 1960–1969; 1970–1979; 1980–1989; 1990–1999; or immigrated in 2000 or later. Whether English was spoken at home (yes, no) was followed by a question for those who did not speak English at home on how well they spoke English (very well, well, not well, not at all). These two questions were combined to derive an *English skill variable* (speaks English at home, speaks English very well, well, not well, not at all). *Citizenship* was dichotomized as yes/no.

### 2.3. Data Analysis

Descriptive analyses compared the six ethnic/immigrant status groups (Ethnicity 2 variable, taking nativity into account) according to functional limitations and demographic characteristics using chi-square statistics. All subsequent logistic regression analyses used functional limitations as the outcome variable and include age, gender, education and family poverty level as control variables. In the first logistic regression of the complete subsample ethnicity 1 was used as an independent variable. In the second logistic regression, ethnicity 2 (ethnicity and nativity) was used as the independent variable of interest. In the third set of analyses three separate logistic regressions were run for each of the three immigrant subsamples (Non-Hispanic White American Immigrants, Mexican American Immigrants and Cuban Immigrants) and decade of immigration was the independent variable of interest. Non-immigrants were excluded from this analysis. The fourth logistic regression analysis was restricted to Mexican Americans born in the U.S. and the key independent variable was whether English was spoken at home. The fifth logistic regression analysis was restricted to immigrants from all three ethnicities and examined the independent variable Ethnicity 1. This analysis added decade of immigration and citizenship to the usual control variables.

## 3. Results

Table 1 presents the prevalence of functional limitations among older adults aged 55–60 and the socio-demographic characteristics of each of the six groups included in the study: non-Hispanic Whites born in U.S., non-Hispanic White immigrants, Mexican Americans born in U.S., Mexican American immigrants, Cuban Americans born in U.S., Cuban American immigrants. White immigrants (22.7%), Mexican American immigrants (24.8%) and non-Hispanic U.S. born Whites (25.5%) had the lowest prevalence of functional limitations. Mexican Americans born in the U.S. (30.1%) and Cuban Americans both immigrants (30.1%) and U.S. born (32.2%) had higher prevalence of functional limitations.

Mexican Americans and Cuban Americans, both those born in the U.S. and immigrants, were more likely to be in the youngest age cohort (55 to 64) than were Non-Hispanic Whites. The majority of all six groups were women. A higher proportion of Mexican American immigrants (66.4%) received only a primary school education compared to other immigrant groups (White 15.2%; Cuban-American 30%) and compared to all U.S. born groups: Whites 6%; Mexican Americans 26.6%; Cuban-Americans 17.8%).

Among non-Hispanic Whites, immigrants had only slightly higher poverty rates than American born (10.9%*versus* 7.2%). Mexican Americans born in the U.S. had a higher prevalence of poverty (14.2%) than U.S. born Whites (7.2%) but lower rates than Mexican American immigrants (20.0%). Cubans had similar rates of poverty regardless of nativity (19%).

Sixty-one percent of non-Hispanic White immigrants and half of the Cuban immigrants arrived before 1970. In contrast, only 36% of Mexican immigrants arrived before 1970. 

Very few Mexican American immigrants or Cuban American immigrants spoke English at home (3.9% and 4.7% respectively). In contrast, 41.1% of non-Hispanic White immigrants spoke English at home. One-quarter of Cuban immigrants (26.6%) and one third of Mexican immigrants (35.7%) spoke no English. Only one in 20 (5.2%) non-Hispanic White immigrants spoke no English. One in ten (10.6%) Cuban Americans born in the U.S. spoke no English. The citizenship rate of non-Hispanic White immigrants and Cuban immigrants were comparable (77%). Less than half of the Mexican Americans were citizens (46.2%). 

**Table 1:** Socio-demographic and immigration-related factors associated with functional limitations among older adults identified by ethnicity and immigrant status (unweighted sample size and weighted %).

	Non-Hispanic White American		Mexican American		Cuban American	
	Born in U.S.	Immigrants	Born in U.S.	Immigrants	Born in U.S.	Immigrants
**Functional Limitation Status**						
No	473,081 (74.5%)	24,495 (77.3%)	9,238 (69.9%)	8,448 (75.2%)	225 (67.8%)	2,646 (69.9%)
Yes	156,260 (25.5%)	6,669 (22.7%)	3,929 (30.1%)	2,930 (24.8%)	89 (32.2%)	1,084 (30.1%)
**Age Group**						
55–64	278,092 (44.9%)	11,774 (38.8%)	6,379 (49.4%)	6,525 (57.4%)	148 (44.8%)	1,220 (33.9%)
65–74	177,237 (27.1%)	9,670 (30.3%)	3,748 (27.2%)	3,097 (26.9%)	82 (25.8%)	1,288 (33.5%)
75–84	126,370 (20.0%)	6,999 (22.1%)	2,471 (19.1%)	1,357 (12.0%)	58 (20.9%)	946 (24.7%)
85+	47,642 (8.0%)	2,721 (8.7%)	569 (4.4%)	399 (3.6%)	26 (8.5%)	276 (7.8%)
**Gender**						
Male	288,097 (45.4%)	12,975 (41.9%)	6,096 (46.6%)	5,425 (48.3%)	143 (44.7%)	1,651 (44.8%)
Female	341,244 (54.6%)	18,189 (58.1%)	7,071 (53.4%)	5,953 (51.7%)	171 (55.3%)	2,079 (55.2%)
**Education**						
Primary	37,011 (6.0%)	4,321 (15.2%)	3,311 (26.6%)	7,475 (66.4%)	42 (17.8%)	1,058 (30.0%)
High school (no diploma)	64,703 (10.2%)	3,441 (10.6%)	2,181 (16.2%)	1,177 (10.0%)	31 (10.3%)	524 (12.9%)
High school diploma	372,282 (58.7%)	14,817 (47.1%)	6,423 (47.9%)	2,260 (19.6%)	155 (44.6%)	1,397 (38.0%)
Bachelors Degree	86,643 (14.1%)	4,033 (13.2%)	707 (5.3%)	280 (2.4%)	51 (16.2%)	416 (11.1%)
Graduate Degree	68,702 (11.1%)	4,552 (13.8%)	545 (4.0%)	186 (1.5%)	35 (11.0%)	335 (8.1%)
**Family Poverty Level**						
Under poverty line	41,721 (7.2%)	2,896 (10.9%)	1,733 (14.2%)	2,175 (20.0%)	52 (18.6%)	638 (18.8%)
100–199%	103,250 (16.7%)	5,337 (18.3%)	2,997 (23.2%)	3,514 (31.3%)	58 (19.1%)	918 (26.5%)
200–299%	105,588 (16.9%)	4,718 (15.6%)	2,444 (19.2%)	2,418 (22.2%)	38 (12.1%)	651 (16.8%)
300–399%	88,773 (14.2%)	3,838 (12.3%)	1,772 (13.8%)	1,380 (12.1%)	50 (17.4%)	467 (12.5%)
400–499%	69,721 (11.2%)	3,114 (10.0%)	1,302 (10.1%)	759 (6.3%)	23 (7.5%)	292 (8.1%)
500% or more	204,150 (33.8%)	10,585 (32.8%)	2,612 (19.5%)	994 (8.2%)	83 (25.4%)	683 (17.3%)
**Immigration Date**						
Born in U.S.	627,007 (99.6%)	0 (0%)	12,914 (98.0%)	0 (0%)	221 (62.4%)	0 (0%)
Before 1950	1,216 (0.2%)	5,098 (15.3%)	68 (0.5%)	655 (5.2%)	8 (1.6%)	76 (1.7%)
1950–1959	688 (0.1%)	8,270 (24.8%)	65 (0.5%)	1,345 (10.6%)	14 (4.2%)	391 (9.6%)
1960–1969	253 (0%)	6,849 (21.2%)	45 (0.4%)	2,442 (20.0%)	26 (10.8%)	1,582 (40.0%)
1970–1979	76 (0%)	4,160 (13.6%)	35 (0.3%)	3,140 (27.3%)	13 (6.1%)	600 (15.7%)
1980–1989	43 (0%)	2,722 (9.4%)	19 (0.2%)	1,720 (15.7%)	19 (8.7%)	529 (15.6%)
1990–1999	43 (0%)	2,837 (10.8%)	11 (0.1%)	1,348 (13.3%)	13 (6.1%)	375 (11.3%)
2000–2006	15 (0%)	1,228 (4.9%)	10 (0.1%)	728 (7.9%)	0 (0%)	177 (6.2%)
**English skill **						
Speaks English at home	612,194 (97.2%)	13,901 (41.1%)	3,692 (28.5%)	496 (3.9%)	129 (37.3%)	179 (4.7%)
Very well	13,684 (2.3%)	8,493 (26.5%)	6,387 (48.6%)	1,520 (12.6%)	102 (29.8%)	844 (21.8%)
Well	2,199 (0.3%)	4,568 (15.1%)	1,971 (13.7%)	2,063 (16.3%)	31 (9.4%)	771 (19.6%)
Not well	1,198 (0.2%)	3,081 (12.0%)	806 (6.4%)	3,490 (31.4%)	30 (12.9%)	1,034 (27.3%)
Not at all	66 (0%)	1,121 (5.2%)	311 (2.9%)	3,809 (35.7%)	22 (10.6%)	902 (26.6%)
**Citizenship**						
Yes	629,341 (100%)	24,311 (77.0%)	13,167 (100.0%)	5,698 (46.2%)	314 (100%)	3,004 (77.1%)
No	0 (0%)	6,853 (23.0%)	0 (0%)	5,680 (53.8%)	0 (0%)	726 (22.9%)

Table 2 combined immigrants and non-immigrants. In comparison to non-Hispanic Whites, both Mexican Americans and Cuban Americans had statistically significant elevated odds of functional limitations when adjustments were made for age and sex. The opposite was observed after further adjustments were made for education level and family poverty: Mexican Americans (OR = 0.76; 95% CI = 0.74–0.78) and Cuban Americans (OR = 0.90; 95% CI = 0.85–0.96) had significantly lower odds of functional limitations compared to non-Hispanic Whites. 

Table 3 shows the odds of functional limitations for each of the six groups, classified by ethnicity and immigration status. In comparison to non-Hispanic Whites born in the U.S., neither Mexican Americans born in the U.S., nor Cuban Americans born in the U.S. differed significantly. However, all three immigrant groups had significantly lower odds of functional limitation status in comparison to non-Hispanic Whites born in the U.S. controlling for age, sex, education and income. 

Table 4 was restricted to immigrant groups only. In comparison to immigrants who arrived since the year 2000, a clear gradient was evident for Mexican Americans who arrived before 1990. Immigrants from the 1980s had 41% higher odds of functional limitations. Among those born before 1950 the odds of functional limitations were more than two times higher. No clear gradient was apparent for non-Hispanic White Americans or Cuban Americans, although the odds of functional limitations were lower among post-millennium immigrants. For both non-Hispanic White immigrants and Mexican immigrants, the odds for functional limitations were significantly higher for those who could not speak English compared to those who spoke English at home. American citizenship was associated with higher odds of functional limitations among non-Hispanic White and Mexican American immigrants.

Table 5 explored the role of language acculturation and functional limitation status among Mexican Americans born in the U.S. Mexican Americans who did not speak English at home had 13% higher odds of functional limitations than those who spoke English at home.

Both Table 6, Table 7 were restricted to immigrants. In comparison to Mexican Americans, when age, sex, education and income were controlled, non-Hispanic White immigrants had comparable odds of functional limitations, and Cuban Americans had higher odds (See Table 6). Further controlling for decade of immigration and citizenship status did not alter these findings.

Table 7 investigated the relationship between age at immigration (childhood, late teens, 20 and over) and functional limitation status within each ethnic group. In comparison to those who had immigrated as adults (age 20 or older), those who immigrated as children had higher odds of functional limitations if they were Mexican American (OR = 1.62, 95% CI = 1.37, 1.92) or Non-Hispanic White immigrants (OR = 1.14; 95% CI = 1.04, 1.24) but lower odds of functional limitations if they were Cuban American (OR = 0.57).

**Table 2:** Logistic regression analyses of functional limitations status by ethnicity (irrespective of immigrant status) for individuals aged 55 and over.

	Model 1 (Adjusted for ethnicity, age & sex)		Model 2 (Model 1 + education & income)	
Variable Name	**Odds Ratio**	**95% C.I.**	**Odds Ratio**	**95% C.I.**
**Ethnicity**				
Non-Hispanic White Americans	1.00	Referent	1.00	Referent
Mexican Americans	1.34	(1.30, 1.37)	0.76	(0.74, 0.78)
Cuban Americans	1.27	(1.20, 1.35)	0.90	(0.85, 0.96)
**Age**				
55–64	1.00	Referent	1.00	Referent
65–74	1.49	(1.47, 1.51)	1.28	(1.26, 1.29)
75–84	2.67	(2.63, 2.71)	2.02	(1.99, 2.05)
85+	5.88	(5.76, 6.01)	4.28	(4.18, 4.37)
**Gender**				
Male	1.00	Referent	1.00	Referent
Female	1.18	(1.17, 1.19)	1.06	(1.05, 1.07)
**Education**				
Primary	--	--	2.78	(2.70, 2.87)
High school (no diploma)	--	--	2.28	(2.21, 2.35)
High school diploma	--	--	1.63	(1.59, 1.67)
Bachelors degree	--	--	1.06	(1.03, 1.10)
Graduate degree	--	--	1.00	Referent
**Family Poverty level**				
Under poverty line	--	--	3.50	(3.42, 3.58)
100–199%	--	--	2.59	(2.54, 2.64)
200–299%	--	--	1.90	(1.86, 1.93)
300–399%	--	--	1.60	(1.57, 1.63)
400–499%	--	--	1.36	(1.33, 1.40)
500% or more	--	--	1.00	Referent

**Table 3:** Logistic regression analyses of functional limitations status by ethnicity and immigrant status for individuals aged 55 and over.

	Model 1 (Adjusted for ethnicity, age & sex)		Model 2 (Model 1 + education & income)	
Variable Name	**Odds Ratio**	**95% C.I.**	**Odds Ratio**	**95% C.I.**
**Ethnicity**				
Non-Hispanic White Americans born in USA	1.00	Referent	1.00	Referent
Non-Hispanic White American immigrants	0.82	(0.80, 0.84)	0.74	(0.72, 0.76)
Mexican Americans born in USA	1.44	(1.39, 1.49)	0.98	(0.94, 1.01)
Mexican American immigrants	1.21	(1.17, 1.26)	0.56	(0.53, 0.58)
Cuban Americans born in the USA	1.45	(1.17, 1.80)	1.16	(0.93, 1.46)
Cuban American Immigrants	1.24	(1.16, 1.32)	0.85	(0.80, 0.91)
**Age**				
55–64	1.00	Referent	1.00	Referent
65–74	1.49	(1.47, 1.52)	1.28	(1.26, 1.30)
75–84	2.68	(2.64, 2.72)	2.01	(1.98, 2.04)
85+	5.90	(5.78, 6.02)	4.26	(4.17, 4.36)
**Gender**				
Male	1.00	Referent	1.00	Referent
Female	1.18	(1.17, 1.20)	1.06	(1.05, 1.08)
**Education**				
Primary	--	--	2.91	(2.82, 3.00)
High school (no diploma)	--	--	2.26	(2.20, 2.33)
High school diploma	--	--	1.61	(1.57, 1.65)
Bachelors degree	--	--	1.06	(1.03, 1.09)
Graduate degree	--	--	1.00	Referent
**Family Poverty level**				
Under poverty line	--	--	3.52	(3.44, 3.60)
100–199%	--	--	2.59	(2.54, 2.64)
200–299%	--	--	1.90	(1.86, 1.93)
300–399%	--	--	1.60	(1.56, 1.63)
400–499%	--	--	1.36	(1.33, 1.40)
500% or more	--	--	1.00	Referent

**Table 4:** Logistic regression analyses of functional limitations status by decade since immigration for non-Hispanic White American Immigrants, Mexican American Immigrants and Cuban American Immigrants controlling for socio-demographic characteristics, English skills & citizenship status.

	Non-Hispanic White American Immigrants		Mexican American Immigrants		Cuban American Immigrants	
	Odds Ratio	95% C.I.	Odds Ratio	95% C.I.	Odds Ratio	95% C.I.
**Immigration Date**						
Before 1950	1.80	(1.48, 2.19)	2.33	(1.77, 3.07)	1.66	(0.83, 3.34)
1950–1959	1.52	(1.27, 1.82)	2.16	(1.70, 2.74)	1.75	(1.11, 2.76)
1960–1969	1.50	(1.25, 1.79)	1.97	(1.59, 2.44)	1.53	(1.03, 2.27)
1970–1979	1.53	(1.27, 1.83)	1.87	(1.52, 2.29)	1.23	(0.82, 1.86)
1980–1989	1.60	(1.33, 1.92)	1.41	(1.14, 1.75)	1.73	(1.17, 2.55)
1990–1999	1.99	(1.68, 2.36)	1.06	(0.85, 1.32)	1.19	(0.81, 1.77)
2000–2006	1.00	Referent	1.00	Referent	1.00	Referent
**Age**						
55–64	1.00	Referent	1.00	Referent	1.00	Referent
65–74	1.57	(1.44, 1.70)	1.53	(1.37, 1.71)	1.49	(1.21, 1.83)
75–84	2.88	(2.63, 3.14)	2.93	(2.54, 3.37)	2.19	(1.76, 2.74)
85+	6.41	(5.71, 7.19)	5.95	(4.72, 7.50)	4.40	(3.24, 5.97)
**Gender**						
Male	1.00	Referent	1.00	Referent	1.00	Referent
Female	1.22	(1.15, 1.30)	1.44	(1.31, 1.58)	1.23	(1.05, 1.43)
**Education**						
Primary	1.47	(1.29, 1.66)	1.50	(0.94, 2.38)	1.34	(0.94, 1.91)
High school (no diploma)	1.24	(1.08, 1.41)	1.07	(0.66, 1.72)	1.03	(0.70, 1.51)
High school diploma	1.30	(1.17, 1.44)	1.08	(0.68, 1.72)	1.38	(0.99, 1.93)
Bachelors degree	1.16	(1.02, 1.32)	0.73	(0.41, 1.32)	1.20	(0.81, 1.77)
Graduate degree	1.00	Referent	1.00	Referent	1.00	Referent
**Family Poverty level**						
Under poverty line	2.83	(2.55, 3.13)	1.87	(1.51, 2.31)	3.27	(2.46, 4.34)
100–199%	2.11	(1.92, 2.31)	1.36	(1.11, 1.67)	1.49	(1.14, 1.95)
200–299%	1.52	(1.38, 1.67)	1.05	(0.85, 1.30)	1.32	(0.99, 1.76)
300–399%	1.41	(1.27, 1.57)	1.13	(0.89, 1.42)	1.14	(0.83, 1.56)
400–499%	1.30	(1.16, 1.46)	0.82	(0.62, 1.08)	1.26	(0.89, 1.79)
500% or more	1.00	Referent	1.00	Referent	1.00	Referent
**English skill**						
Speaks English at home	1.00	Referent	1.00	Referent	1.00	Referent
Very well	0.97	(0.90, 1.05)	0.77	(0.59, 1.02)	0.87	(0.58, 1.31)
Well	1.21	(1.10, 1.33)	0.87	(0.67, 1.13)	0.88	(0.59, 1.32)
Not well	2.05	(1.85, 2.27)	1.18	(0.92, 1.51)	1.23	(0.84, 1.81)
Not at all	3.45	(2.99, 2.98)	1.34	(1.04, 1.73)	1.36	(0.92, 2.02)
**Citizenship**						
Yes	1.18	(1.09, 1.28)	1.12	(1.01, 1.24)	0.94	(0.74, 1.18)
No	1.00	Referent	1.00	Referent	1.00	Referent

**Table 5:** Logistic regression analyses of functional limitations status by language spoken at home and socio-demographic characteristics among U.S. born Mexican Americans aged 55 and over.

	Odds Ratio	(95% C.I.)
**Ethnicity**		
Speaks English at home	1.00	Referent
Does not Speak English at home	1.13	(1.03, 1.24)
**Age**		
55–64	1.00	Referent
65–74	1.19	(1.08, 1.31)
75–84	2.04	(1.83, 2.27)
85+	4.23	(3.48, 5.14)
**Gender**		
Male	1.00	Referent
Female	1.13	(1.04, 1.22)
**Education**		
Primary	2.49	(1.86, 3.33)
High school (no diploma)	2.02	(1.50, 2.71)
High school diploma	1.62	(1.22, 2.15)
Bachelors degree	1.31	(0.93, 1.85)
Graduate degree	1.00	Referent
**Family Poverty level**		
Under poverty line	3.10	(2.65, 3.62)
100–199%	2.54	(2.20, 2.94)
200–299%	1.79	(1.54, 2.07)
300–399%	1.36	(1.16, 1.60)
400–499%	1.15	(0.96, 1.38)
500% or more	1.00	Referent

**Table 6:** Logistic regression analyses of functional limitations status for Mexican American immigrants, Cuban American Immigrants & non-Hispanic White immigrants aged 55 and over.

	Model 1 (Adjusted for ethnicity, age & sex)		Model 2 (Model 1 + education & income)		Model 3 (Model 2 + decade since immigration)	
	Odds Ratio	95% C.I.	Odds Ratio	95% C.I.	Odds Ratio	95% CI
**Ethnicity**						
Non-Hispanic White American immigrants	0.63	(0.59, 0.66)	0.99	(0.93, 1.05)	0.97	(0.91, 1.04)
Mexican American immigrants	1.00	Referent	1.00	Referent	1.00	Referent
Cuban American immigrants	0.96	(0.88, 1.04)	1.23	(1.13, 1.35)	1.18	(1.08, 1.30)
**Age**						
55–64	1.00	Referent	1.00	Referent	1.00	Referent
65–74	1.80	(1.70, 1.91)	1.62	(1.53, 1.72)	1.66	(1.56, 1.76)
75–84	3.66	(3.44, 3.89)	3.04	(2.86, 3.24)	3.12	(2.91, 3.33)
85+	7.77	(7.14, 8.45)	6.54	(6.00, 7.13)	6.70	(6.10, 7.37)
**Gender**						
Male	1.00	Referent	1.00	Referent	1.00	Referent
Female	1.37	(1.31, 1.44)	1.29	(1.23, 1.36)	1.30	(1.23, 1.36)
**Education**						
Primary	--	--	1.84	(1.65, 2.04)	1.91	(1.71, 2.12)
High school (no diploma)	--	--	1.22	(1.09, 1.37)	1.28	(1.14, 1.44)
High school diploma	--	--	1.30	(1.18, 1.43)	1.34	(1.22, 1.48)
Bachelors degree	--	--	1.18	(1.05, 1.33)	1.18	(1.04, 1.33)
Graduate degree	--	--	1.00	Referent	1.00	Referent
**Family Poverty level**						
Under poverty line	--	--	3.14	(2.89, 3.41)	3.08	(2.83, 3.35)
100–199%	--	--	2.09	(1.94, 2.26)	2.07	(1.92, 2.24)
200–299%	--	--	1.51	(1.39, 1.64)	1.51	(1.39, 1.64)
300–399%	--	--	1.45	(1.32, 1.59)	1.44	(1.31, 1.58)
400–499%	--	--	1.28	(1.15, 1.42)	1.27	(1.14, 1.40)
500% or more	--	--	1.00	Referent	1.00	Referent
**Immigration Date**						
Before 1950	--	--	--	--	1.15	(1.01, 1.32)
1950–1959	--	--	--	--	1.05	(0.93, 1.19)
1960–1969	--	--	--	--	1.15	(1.02, 1.30)
1970–1979	--	--	--	--	1.27	(1.12, 1.43)
1980–1989	--	--	--	--	1.31	(1.15, 1.48)
1990–1999	--	--	--	--	1.55	(1.37, 1.75)
2000–2006	--	--	--	--	1.00	Referent
**Citizenship**						
Yes	--	--	--	--	1.18	(1.11, 1.25)
No	--	--	--	--	1.00	Referent

**Table 7:** Logistic regression analyses of functional limitations status by age at immigration for non-Hispanic White American immigrants, Mexican American immigrants and Cuban American Immigrants controlling for socio-demographic characteristics, English skills & citizenship status.

	Non-Hispanic White American Immigrants (n = 30,488)		Mexican American Immigrants (n = 11,240)		Cuban American Immigrants (n = 3,649)	
	Odds Ratio	95% C.I.	Odds Ratio	95% C.I.	Odds Ratio	95% C.I.
**Age at Immigration **						
Under 16	1.14	(1.04, 1.24)	1.62	(1.37, 1.92)	0.57	(0.37, 0.88)
16–19	1.09	(0.97, 1.22)	1.14	(0.95, 1.36)	1.05	(0.71, 1.53)
20 and over	1.00	Referent	1.00	Referent	1.00	Referent
**Age**						
55–64	1.00	Referent	1.00	Referent	1.00	Referent
65–74	1.58	(1.46, 1.71)	1.63	(1.46, 1.81)	1.37	(1.11, 1.69)
75–84	3.04	(2.80, 3.30)	3.26	(2.86, 3.72)	2.07	(1.66, 2.59)
85+	6.89	(6.21, 7.66)	6.63	(5.32, 8.26)	4.20	(3.09, 5.70)
**Gender**						
Male	1.00	Referent	1.00	Referent	1.00	Referent
Female	1.23	(1.15, 1.30)	1.40	(1.28, 1.54)	1.21	(1.04, 1.42)
**Highest Level of Education**						
Primary	1.41	(1.24, 1.59)	1.62	(1.02, 2.56)	1.42	(1.00, 2.02)
Some high school	1.20	(1.05, 1.37)	1.10	(0.68, 1.77)	1.08	(0.74, 1.58)
High school diploma	1.27	(1.14, 1.41)	1.12	(0.70, 1.78)	1.45	(1.04, 2.02)
Bachelors degree	1.16	(1.02, 1.32)	0.74	(0.41, 1.32)	1.19	(0.81, 1.76)
Graduate degree	1.00	Referent	1.00	Referent	1.00	Referent
**Family Poverty level**						
Under poverty line	2.86	(2.59, 3.17)	1.84	(1.49, 2.27)	3.12	(2.35, 4.13)
100–199%	2.12	(1.93, 2.32)	1.36	(1.11, 1.67)	1.42	(1.09, 1.86)
200–299%	1.52	(1.38, 1.68)	1.05	(0.84, 1.29)	1.23	(0.91, 1.64)
300–399%	1.41	(1.27, 1.57)	1.14	(0.91, 1.44)	1.06	(0.77, 1.46)
400–499%	1.31	(1.16, 1.47)	0.82	(0.62, 1.09)	1.24	(0.87, 1.76)
500% or more	1.00	Referent	1.00	Referent	1.00	Referent
**English skill**						
Not at all	1.00	Referent	1.00	Referent	1.00	Referent
Speaks English at home	0.28	(0.25, 0.32)	0.78	(0.61, 1.00)	0.82	(0.56, 1.22)
Very well	0.27	(0.24, 0.31)	0.62	(0.51, 0.75)	0.75	(0.57, 0.99)
Well	0.35	(0.30, 0.39)	0.73	(0.62, 0.85)	0.69	(0.54, 0.89)
Not well	0.60	(0.53, 0.68)	0.96	(0.86, 1.08)	0.94	(0.76, 1.15)
**Citizenship**						
Yes	1.25	(1.16, 1.35)	1.30	(1.17, 1.44)	1.09	(0.89, 1.33)
No	1.00	Referent	1.00	Referent	1.00	Referent

## 4. Discussion

Although much attention has been focused on the existence of an “Hispanic Paradox,” most of the research on this paradox has focused on mortality rates or discrete health outcomes (e.g., cardiovascular disease) rather than conditions such as later-life functional limitations which also have important implications for health and well-being. In the U.S., such research is particularly salient as Hispanics constitute not only the largest immigrant population but also one of the least advantaged from the perspective of income, education and related factors.

This study sought to determine whether or not the commonly observed Hispanic Paradox that exists for mortality [9,34] is also observed for functional limitations among older adults in the U.S., and if so, whether any of the leading explanations of the paradox (healthy lifestyles, healthy migrant, and reverse migration hypotheses) help explain patterns of functional limitations among older adults. We used a large nationally representative data set, the U.S. Census Bureau’s American Community Survey, to examine trends among immigrant and U.S. born Mexican Americans, Cuban Americans, and non-Hispanic White Americans. 

Our data supported the existence of an Hispanic paradox with respect to functional limitations among older adults in the U.S. Below, we summarize our study findings and discuss the implications of our findings in light of existing literature on the Hispanic paradox.

(1)* Does an Hispanic Paradox exist for functional limitations?* Our data supported the existence of an Hispanic Paradox with respect to functional limitations when socioeconomic position (SEP) was taken into account. In order to determine whether the Hispanic Paradox existed among older adults with respect to functional limitations, we examined whether Mexican and/or Cuban Americans had better (*i.e.*, lower) odds of functional limitations than non-Hispanic white Americans when adjusted for age, sex, income and education. Both Mexican and Cuban Americans had *higher* odds of functional limitations (OR = 1.34 and 1.27 respectively) when ethnicity, age and sex were controlled (Table 2). 

However, additional adjustments for education and income resulted in significantly lower odds of functional limitations among Mexican and Cuban Americans compared to non-Hispanic White Americans (OR = 0.76 and 0.90, respectively). Thus, our findings of a SEP-adjusted Hispanic advantage in functional limitations are in keeping with those on the Hispanic mortality advantage [9]. 

(2) *Healthy lifestyle explanation.* The Hispanic lifestyle hypothesis suggests it is the health behaviors and other lifestyle factors such as social support of Hispanic Americans (in our study, both Mexican Americans and Cuban Americans) that are the primary reason for the relatively good health outcomes. Thus, Hispanic Americans, whether immigrants or U.S. born, should have lower SEP-adjusted odds of functional limitations than non-Hispanic Whites. It is probable that Hispanics born in the US would retain some aspects of the healthy Hispanic lifestyle, though probably to a lesser extent than immigrant groups and therefore the health benefits for U.S. born Hispanics would be more modest than that of immigrant Hispanics but still significantly better than that of non-Hispanic Whites. This hypothesis was partially supported: Both Mexican American immigrants and Cuban immigrants had lower SEP-adjusted odds of functional limitations than non-Hispanic Whites born in the U.S. In contrast to the theory, neither U.S. born Hispanic group differed significantly from non-Hispanic Whites born in the U.S. when age, sex, income and education level were controlled (Table 3). When only immigrants were included in the analysis (Table 6), non-Hispanic White immigrants had SEP-adjusted odds of functional limitations that were comparable to Mexican American immigrants and Cuban Americans had higher odds of functional limitations. This finding is also incongruent with the original hypothesis that Hispanic status should be protective with respect to disability outcomes. These findings suggest that it may be something related to immigration rather than Hispanic ethnicity, *per se*, affecting functional limitations among older U.S. adults. 

To further test the healthy migrant hypothesis, we examined whether acculturation moderated the association between ethnicity and functional limitations. If Hispanic lifestyle is indeed protective, those who are least acculturated should have the lowest levels of functional limitations when controlling for other factors. Using a common measure of acculturation (language use at home), we hypothesized that Hispanic individuals who spoke English at home should be more acculturated than those who spoke Spanish at home. This finding should be true for both Hispanic immigrants and those born in the U.S. Those who speak no English at home should be the least acculturated and thus the healthiest once adjustments have been made for SES.

To the contrary, our findings showed that Mexican immigrants who did not speak any English had significantly *higher* odds of functional limitations than immigrants who spoke English at home (See Table 4). Cuban immigrants who did not speak English also had elevated odds of disability in comparison to those who spoke English at home, although these odds failed to reach statistical significance (OR = 1.36; 95% CI = 0.92−2.02). Similarly, among Mexican Americans born in the U.S., those who did not speak English at home had higher SEP-adjusted odds of functional limitations than those who spoke English at home (See Table 5). Although these findings are in contrast to the findings expected if the healthy Hispanic lifestyle explanation was correct, the results are consistent with earlier research [5] suggesting that for older Hispanic immigrants, acculturation is associated with better health outcomes in areas such as functional activities and cognitive functioning scores. 

(3) *Healthy migrant explanation.* According to this hypothesis, it is migration itself, not ethnicity, which is important for health. Therefore, not only Hispanic immigrants but also non-Hispanic White immigrants should have better SEP-adjusted rates of functional limitations than non-Hispanic Whites born in the U.S. This theory received substantial support in the analyses. In Table 3, all immigrants groups, including non-Hispanic White immigrants, had statistically lower odds of functional limitations than non-Hispanic Whites born in the U.S. when age, gender, education and family income were adjusted.

If the Hispanic paradox is due primarily to the healthy migrant effect, the hypothesis suggests U.S. born Hispanics and non-Hispanic Whites, regardless of ethnicity, should have comparable rates of functional limitations. In support of this hypothesis, both Cuban Americans born in the U.S. and Mexican Americans born in the U.S. had comparable SEP-adjusted odds of functional limitations in comparison to non-Hispanic Whites born in the U.S.

According to the healthy migrant explanation, the protective effect of self-selection and health screening of new immigrants would decrease over time as immigrants acquire diseases and face acculturation stress in the post immigration period [23]. In our study, support for this hypothesis was mixed. Among Mexican Americans, there was a dose-response relationship, such that the longer individuals had been in the U.S., the worse their SEP-adjusted disability rates (Table 4). Among non-Hispanic White immigrants, those who had been in the U.S. for a long duration had significantly higher odds of functional limitations than those who arrived since 2000, but the relationship was not a clear or consistent gradient. Among Cuban immigrants, individuals who had immigrated in each decade preceding the year 2000 had higher odds of functional limitations than those who arrived in the new millennium. However, there was not a clear gradient and only three of the decades reached statistical significance.

We believed an examination of age at immigration would allow us greater insight into the potential role of self-selection. We posit that positive self-selection would not be as evident for those who migrated as children, where the process of migration can be assumed to be instigated by the parents or guardians rather than the respondent. Our findings were mixed. We found that the odds of functional limitations were significantly higher for non-Hispanic Whites and Mexican Americans who immigrated before the age of 16, in comparison to those who immigrated as adults. However, Cuban Americans who immigrated when they were young had lower odds of functional limitations than those who immigrated as adults. This discrepancy may be due to differences in immigration history: older Cubans often came as refugees as opposed to older Mexican Americans who came as immigrants. It is possible that the positive health selection effects are disproportionately found among immigrants who pro-actively sought new challenges in a new land rather than refugees who were forced to flee their homeland.

(4) *Reverse migration or* “*Salmon Effect*” *explanation.* This hypothesis theorizes that Mexican Americans immigrants who are ill or disabled are more likely than Cuban Americans immigrants to return to their country of origin. An interesting natural experiment has occurred which makes it easier for us to examine this theory. As noted earlier, at this writing, it remains very unlikely that ill Cuban Americans would return to their country of origin due to the political situation in that country and travel constraints. Further, as Rumbaut *et al.* [38] point out, the more favorable reception of and attitude toward Cuban Americans in the U.S. than toward Mexican Americans may make staying through old age a more positive experience for Cubans Americans. Comparing Cuban Americans and Mexican Americans therefore allows us to assess the validity of the reverse-migration explanation. 

If reverse migration explains a major portion of the Hispanic Paradox then Cuban Americans immigrants should have higher odds of functional limitations when compared to Mexican American immigrants. Furthermore, Mexican American immigrants should have lower SEP-adjusted odds of disability when compared to non-Hispanic Whites born in the U.S. Consistent with this hypothesis, Cuban American immigrants had higher odds of disability than Mexican American immigrants (Table 6) and Mexican American immigrants had lower SEP-adjusted odds of disability when compared to non-Hispanic Whites born in the U.S. (Table 3). If reverse migration by Hispanic immigrants is the major cause of the Hispanic paradox, then Mexican Americans and Cuban Americans born in the U.S. should have comparable SEP-adjusted disability rates to non-Hispanic Whites born in the U.S. once other important factors, such as socio-demographic differences, were adjusted. Table 3 provides support for this hypothesis. Neither group of U.S.-born Hispanics differed significantly from non-Hispanic Whites born in the U.S. when adjustments for income and education were made. In contrast to our findings for functional limitations, Crimmins’ study [22] found that U.S.-born Mexican Americans had worse total biological risk profiles than did non-Hispanic Whites even after adjustments for socioeconomic status. The discrepancy between the findings of Crimmins’ study and our study may be due to the different outcomes evaluated in the two studies. 

The reverse migration hypothesis also suggests that Mexican Americans immigrants are less likely to return to their country of origin (*i*) the longer they have resided in the U.S. and (*ii*) if they have citizenship and the rights it entails (e.g., access to government-sponsored health insurance for low income citizens such as Medicaid). These trends should not be apparent for the Cuban American immigrants because they are far less likely to have the option of returning to their country of origin.

These hypotheses were supported, with a dose-response relationship observed for Mexican Americans (Table 4). Those who arrived before 1950 had more than twice the odds of functional limitations than those who arrived since 2000. Among Cuban Americans, the odds of functional limitations were higher for those who arrived before 2000 but only some of the decades reach statistical significance, and no clear gradient was found. 

In keeping with the reverse migration hypothesis, American citizenship was associated with higher odds of functional limitations for Mexican American immigrants and non-Hispanic White immigrants but not for Cuban immigrants (Table 7). Again, it is important to remember that due to the political climate in Cuba, few Cubans return home, whether or not they have citizenship status; this alone may explain the lack of a significant association between citizenship and functional limitations among Cubans. 

Recent research on the Hispanic paradox in mortality suggest that although there is some support for a reverse migration effect, the magnitude of the effect is modest and could not explain the majority of the mortality difference between Hispanics and non-Hispanic Whites [34]. Our findings for functional limitations similarly suggest a significant role for reverse migration but there exists a residual Hispanic advantage that was not explained by reverse migration.

### Limitations

Several limitations should be considered in interpreting the study findings. First, we could not identify through this data set the reasons for functional limitations and whether they were from cumulative wear on the body or secondary to a condition such as heart disease or diabetes. We also relied on self-report of functional limitations which has been shown to vary with the timing of the assessment as well as other factors [39]. If there were ethnic differences in the perception of disability, our findings may reflect a combination of these differences with true variation in disability in the population. The sample size of U.S.-born older Cuban Americans was much smaller than the other groups examined, but with 314 respondents there was sufficient power for the analyses conducted. Our inability to measure health behaviors using the ACS data set limited our ability to draw firm conclusions regarding the healthy lifestyle hypothesis. A more direct test of this hypothesis would include several important health behaviors known to differ between U.S. born and foreign-born Hispanics such as smoking, eating behavior, and physical activity. To more fully understand the healthy migrant effect and the reverse migration effect, future studies of the Hispanic paradox would benefit from the use of surveys that followed immigrants from the source country to the U.S. and then track return migrations. Last, the healthy lifestyle hypothesis was tested using English language use as a measure of acculturation. Although a common measure of acculturation, English language use is just one of many aspects of the acculturation or assimilation process. Future studies should use a fuller complement of measures capturing acculturation patterns. 

Despite these limitations, however, the findings of this study shed new light on the complex issue of the Hispanic Paradox, both in applying it to the understudied topic of functional limitations in later life and in including Cuban Americans in the sample.

## 5. Conclusions

This study provides support for the existence of an Hispanic Paradox with respect to functional limitations in later life, as well as clear support for the healthy migrant hypothesis for Mexican, Cuban, and White immigrants. 

The results of our study also show partial support for reverse migration, with citizenship and longer duration in the USA associated with higher rates of SEP-adjusted disability for Mexican Americans. However, if the Hispanic Paradox was due solely or primarily to the reverse migration effect, Cuban American immigrants, who typically cannot return to their country of origin, should have disability rates comparable to Whites born in the U.S. They did not, but rather had significantly lower SEP-adjusted odds of functional limitations in comparison to Whites born in the USA.

The Hispanic healthy life-style explanation had little support in this study, with English language use at home, a marker for acculturation, associated with *better*, not poorer, functional limitations outcomes for both the Mexican American immigrant and non-immigrant respondents. Furthermore, neither U.S.-born Mexican Americans nor Cuban Americans differed significantly in disability outcomes with respect to non-Hispanic Whites born in the U.S. As noted above, however, important new data on smoking in relation to the Hispanic Paradox suggests the need for further teasing apart of this particular lifestyle factor, which also is known to be strongly associated with functional limitations in later life [40]. Since, the prevalence of smoking is similar among immigrant and non-immigrant Mexican Americans [22], smoking cannot explain the difference between these two groups in disability outcomes.

In sum, the Hispanic Paradox with respect to functional limitations for older Mexican Americans appears to be due to a combination of self-selection of healthy immigrants (healthy migrant effect) and reverse migration of some ill and/or disabled migrants (Salmon or unhealthy out-migration effect).

Future research should focus on improving our understanding the process of self-selection for those who immigrate to the US and (in the case of Mexican Americans) those who choose to leave the U.S. A better understanding of self-section may help to clarify the Hispanic Paradox by identifying what factors influence better health outcomes in those who migrate to the U.S. Likewise, it is important to look at those who were unsuccessful in immigrating. These avenues for future research should better inform our understanding of the dynamic interplay of barriers to and facilitators of successful immigration, and how these factors influence long-term health and disability outcomes.

Our findings have wider implications for future long-term policy for the elderly. Despite these data’s support for the Hispanic Paradox when income and education are accounted for, it is important to note that the unadjusted prevalence of functional limitations among older Hispanics is disturbingly high, ranging from one in four (for Mexican American immigrants) to one in three (for Cuban Americans born in the U.S.). With the demographic growth of the Hispanic population, healthcare policy must be attentive to such findings, and the likely greater preventive and health care needs of a large older Hispanic population. Other findings of this study point to the need for increased bilingual services with the aging of the Hispanic population for whom limited English is strongly associated with functional limitations. Although continuing economic difficulties in the US and strong anti-immigrant sentiments in a sizable part of the voting population may make such expansions politically unlikely over the short term, policies that increase access to healthcare and community support would be beneficial [41]. With the demonstrated desire among an overwhelming majority of Americans to “age in place” rather than in institutional settings [39], and our increasing understanding of the value of building communities as a preventive and reactive measure to creating better health outcomes, the need for such policies is underscored.
